# Chloranilic acid: a redetermination at 100 K

**DOI:** 10.1107/S1600536810003387

**Published:** 2010-01-30

**Authors:** Grzegorz Dutkiewicz, H. S. Yathirajan, Q. N. M. Hakim Al-arique, B. Narayana, Maciej Kubicki

**Affiliations:** a Department of Chemistry, Adam Mickiewicz University, Grunwaldzka 6, 60-780 Poznań, Poland; b Department of Studies in Chemistry, University of Mysore, Manasagangotri, Mysore 570 006, India; c Department of Studies in Chemistry, Mangalore University, Mangalagangotri 574 199, India

## Abstract

The crystal structure of chloranilic acid, C_6_H_2_Cl_2_O_4_, was first described by Andersen in 1967 [Andersen, E. K. (1967). Acta Cryst. **22**, 188–191] at room temperature using visually estimated intensities. Taking into account the importance of the title compound, we have redetermined the structure at 100 (1) K. The approximately planar mol­ecule [the maximum deviation from the mean plane through the ring is 0.0014 (9) Å for the ring atoms and 0.029 (3) Å for the other atoms] occupies a special position, lying across the center of symmetry. In the crystal structure, a two-dimensional hydrogen-bonded network sustained by O—H⋯O inter­actions runs approximately parallel to [101]. The two-dimensional layers are further packed in a parallel fashion, stabilized by Cl⋯Cl inter­actions [Cl⋯Cl = 3.2838 (8) Å, C—Cl⋯Cl = 152.96 (6)°].

## Related literature

For charge-transfer complexes of chloranilic acid, see: Gotoh *et al.* (2006 ▶, 2007 ▶, 2008 ▶); Gotoh & Ishida (2009 ▶); Ishida (2004 ▶); Ishida & Kashino (1999 ▶). For a recent study of the formation of either salts or co-crystals by chloranilic acid with different organic bases, see: Molčanov & Kojić-Prodić (2010 ▶). For the previous determination of the title structure, see: Andersen (1967*a* ▶) and of its hydrate, see: Andersen (1967*b* ▶). For hydrogen-bond motifs, see: Bernstein *et al.* (1995 ▶). For a description of the Cambridge Structural Database, see: (Allen, 2002 ▶). 
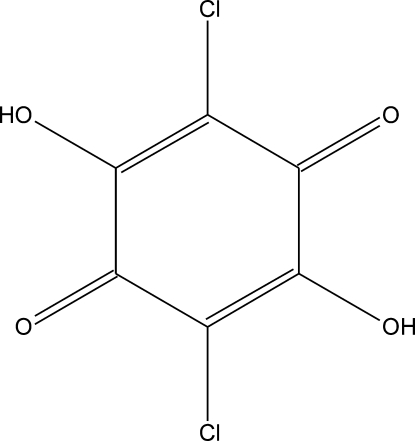


## Experimental

### 

#### Crystal data

C_6_H_2_Cl_2_O_4_
*M* *_r_* = 208.98
Monoclinic, 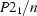

*a* = 7.5338 (12) Å
*b* = 5.5225 (10) Å
*c* = 8.5720 (12) Å
β = 104.868 (11)°
*V* = 344.70 (10) Å^3^
*Z* = 2
Mo *K*α radiation
μ = 0.90 mm^−1^
*T* = 100 K
0.3 × 0.1 × 0.1 mm

#### Data collection

Oxford Diffraction Xcalibur Eos diffractometer
Absorption correction: multi-scan (*CrysAlis PRO*; Oxford Diffraction (2009 ▶)) *T*_min_ = 0.857, *T*_max_ = 1.000
6154 measured reflections
774 independent reflections
698 reflections with *I* > 2σ(*I*)
*R*_int_ = 0.032

#### Refinement

*R*[*F* ^2^ > 2σ(*F* ^2^)] = 0.025
*wR*(*F* ^2^) = 0.056
*S* = 1.09
774 reflections
59 parameters
All H-atom parameters refined
Δρ_max_ = 0.37 e Å^−3^
Δρ_min_ = −0.27 e Å^−3^

### 

Data collection: *CrysAlis PRO* (Oxford Diffraction, 2009 ▶); cell refinement: *CrysAlis PRO*; data reduction: *CrysAlis PRO* program(s) used to solve structure: *SIR92* (Altomare *et al.*, 1993 ▶); program(s) used to refine structure: *SHELXL97* (Sheldrick, 2008 ▶); molecular graphics: *Stereochemical Workstation Operation Manual* (Siemens, 1989 ▶); software used to prepare material for publication: *SHELXL97*.

## Supplementary Material

Crystal structure: contains datablocks I, global. DOI: 10.1107/S1600536810003387/ds2017sup1.cif

Structure factors: contains datablocks I. DOI: 10.1107/S1600536810003387/ds2017Isup2.hkl

Additional supplementary materials:  crystallographic information; 3D view; checkCIF report

## Figures and Tables

**Table 1:** Hydrogen-bond geometry (Å, °)

*D*—H⋯*A*	*D*—H	H⋯*A*	*D*⋯*A*	*D*—H⋯*A*
O2—H2⋯O3^i^	0.82 (2)	2.00 (2)	2.7516 (15)	152 (2)

Symmetry code: (i) 
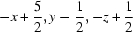
.

**Fig. 1.**
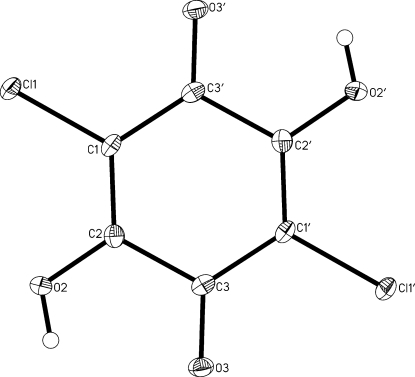
Anisotropic ellipsoid representation of the compound I together with atom labelling scheme. The ellipsoids are drawn at 50% probability level, hydrogen atoms are depicted as spheres with arbitrary radii.

**Fig. 2.**
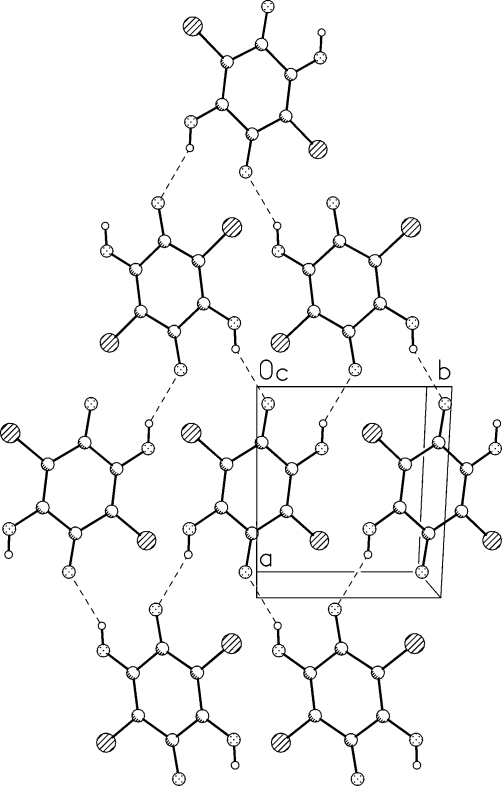
The hydrogen bonded motifs in the crystal structure of I. Hydrogen bonds are shown as dashed lines. (a) the N—H···N chain. Symmetry codex: (i) x,y,z; (ii) x,-3/2+y,3/2-z; (iii) 2-x,1/2+y,3/2-z; (iv) 2-x,-1/2+y,3/2-z; (v) 2-x,-3/2+y,3.2-z. (b) the N—H···O chains and rings. Symetry codes: (i) x,y,z; (ii) 1-x,1/2+y,3/2-z; (iii) 1-x,-1/2+y,3/2-z.

**Fig. 3.**
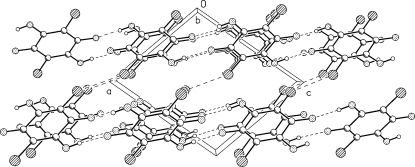
Crystal packing as seen along y-direction. Hydrogen bonds and Cl···Cl contacts are shown as dashed lines.

## supplementary crystallographic information

### Comment

The crystal structures of various charge-transfer complexes of chloranilic acid have been reported (Gotoh, Asaji *et al.*, 2008; Gotoh, Asaji *et al.*, 2007; Gotoh & Ishida, 2009; Gotoh, Ishikawa, *et al.*, 2006; Ishida, 2004; Ishida & Kashino, 1999). Very recently, a study on the formation of either salts or co-crystals by chloranilic acid with the different organic bases was published (Molčanov & Kojić-Prodić, 2010).

There is a number of structures in the Cambridge Database (Allen, 2002) that contain the chloranilic acid (2,5-dichloro-3,6-dihydroxycyclohexa-2,5-diene-1,4-dione, I - Scheme 1), either as a neutral molecule or as an anion (mono- or di-). Interestingly, the only determination of the structure of the acid itself dates back to 1967 (Andersen, 1967a; hereinafter referred to as KA67). The structure was refined based on the visually estimated intensities of the diffraction spots obtained by means of the Weissenberg equi-inclination method. The quality of this structure is excellent taking into account the technology involved, but - having in mind the importance of this small molecule - thanks to the advancement of the methodology it might be desirable to get the more accurate results. Here we report the results of the structure determination of (I) at 100 (1) K. The unit cell parameters of the accompanying room temperature experiment are in an excellent agreement with the data of KA67, but the model is much better, for instance in terms of R factors (8.9% in 1967, with 22 reflections omitted *vs.* 2.5% in the present determination), the only symmetry independent hydrogen atom was found in the difference Fourier map in KA67 and left in the position found, while now it was isotropicaly refined, etc. Nevertheless, the basic features of the structure are similar, and both the precision and depth of the analysis in KA67 and accompanying paper on the hydrate (Andersen, 1967b) are really remarkable.

We have chosen to describe the structure in the P2_1_/n space group instead of P2_1_/a used in KA67, in order to have smaller β angle (104.87° instead of 122.77°); the transformation matrix is {-1 0 -1 0 1 0 1 0 0}. The molecule of I lies in the special position, across the center of symmetry (Z'=1/2). The whole molecule is planar (Fig. 1); the maximum deviation form the mean plane through 6 ring atom is 0.0014 (9) Å for the ring atom and 0.029 (3)Å for the other atoms. The bond length pattern confirms the dominant double-bond character for the bonds C3—O3 (1.224 (2) Å) and C1—C2 (1.349 (2) Å) and single-bond for C2—C3 (1.507 (2) Å) and - to the lesser extent - for C1—C3' (1.450 (2) Å).

In the crystal structure the main packing motif arises as the result of relatively strong intermolecular O—H···O hydrogen bonds, which make the antiparallel chains of molecules related by the 2_1_ screw along y direction; using the graph-set notation (Bernstein *et al.*, 1995), these first-order chains will be described as C(5). The neighboring chains are interconnected to give the centrosymmetric second-order rings R44(22) - cf. Fig. 2. These structures produce the one-molecule thick layers of molecules which expand along [101] direction, and the neighboring chains are connected by means of van der Waals interactions and probably also by weak halogen bonds, with Cl···Cl distance of 3.2838 (8)Å and C—Cl···Cl angle of 152.96 (6)° - Fig. 3.

### Experimental

Chloranilic acid was purchased from Loba Chemie, Mumbai, India. X-ray quality crystals were obtained from methanol solution after slow evaporation.

### Refinement

The position of the hydrogen atom was found in the difference Fourier map and both the positional and isotropic thermal parameters weres freely refined.

### Crystal data

**Table:**

C_6_H_2_Cl_2_O_4_	*F*(000) = 208
*M**_r_* = 208.98	*D*_x_ = 2.014 Mg m^−^^3^
Monoclinic, *P*2_1_/*n*	Mo *K*α radiation, λ = 0.71073 Å
Hall symbol: -P 2yn	Cell parameters from 4539 reflections
*a* = 7.5338 (12) Å	θ = 2.8–27.8°
*b* = 5.5225 (10) Å	µ = 0.90 mm^−^^1^
*c* = 8.5720 (12) Å	*T* = 100 K
β = 104.868 (11)°	Prism, red
*V* = 344.70 (10) Å^3^	0.3 × 0.1 × 0.1 mm
*Z* = 2	

### Data collection

**Table:**

Oxford Diffraction Xcalibur Eos diffractometer	774 independent reflections
Radiation source: Enhance (Mo) X-ray Source	698 reflections with *I* > 2σ(*I*)
graphite	*R*_int_ = 0.032
Detector resolution: 16.1544 pixels mm^-1^	θ_max_ = 27.9°, θ_min_ = 3.2°
ω–scan	*h* = −9→9
Absorption correction: multi-scan (*CrysAlis PRO*; Oxford Diffraction (2009))	*k* = −7→7
*T*_min_ = 0.857, *T*_max_ = 1.000	*l* = −11→10
6154 measured reflections	

### Refinement

**Table:**

Refinement on *F*^2^	Primary atom site location: structure-invariant direct methods
Least-squares matrix: full	Secondary atom site location: difference Fourier map
*R*[*F*^2^ > 2σ(*F*^2^)] = 0.025	Hydrogen site location: inferred from neighbouring sites
*wR*(*F*^2^) = 0.056	All H-atom parameters refined
*S* = 1.09	*w* = 1/[σ^2^(*F*_o_^2^) + (0.0204*P*)^2^ + 0.3502*P*] where *P* = (*F*_o_^2^ + 2*F*_c_^2^)/3
774 reflections	(Δ/σ)_max_ < 0.001
59 parameters	Δρ_max_ = 0.37 e Å^−^^3^
0 restraints	Δρ_min_ = −0.27 e Å^−^^3^

### Special details

**Table:**

Geometry. All esds (except the esd in the dihedral angle between two l.s. planes) are estimated using the full covariance matrix. The cell esds are taken into account individually in the estimation of esds in distances, angles and torsion angles; correlations between esds in cell parameters are only used when they are defined by crystal symmetry. An approximate (isotropic) treatment of cell esds is used for estimating esds involving l.s. planes.
Refinement. Refinement of *F*^2^ against ALL reflections. The weighted *R*-factor wR and goodness of fit *S* are based on *F*^2^, conventional *R*-factors *R* are based on *F*, with *F* set to zero for negative *F*^2^. The threshold expression of *F*^2^ > σ(*F*^2^) is used only for calculating *R*-factors(gt) etc. and is not relevant to the choice of reflections for refinement. *R*-factors based on *F*^2^ are statistically about twice as large as those based on *F*, and *R*- factors based on ALL data will be even larger.

### Fractional atomic coordinates and isotropic or equivalent isotropic displacement parameters (Å^2^)

**Table:**

	*x*	*y*	*z*	*U*_iso_*/*U*_eq_	
C1	0.8496 (2)	−0.1699 (3)	0.47066 (18)	0.0102 (3)	
Cl1	0.66487 (5)	−0.36207 (7)	0.44138 (4)	0.01295 (13)	
C2	0.9798 (2)	−0.1990 (3)	0.38967 (18)	0.0101 (3)	
O2	0.97189 (15)	−0.3741 (2)	0.28296 (13)	0.0132 (3)	
H2	1.064 (3)	−0.381 (4)	0.249 (3)	0.034 (7)*	
C3	1.14042 (19)	−0.0274 (3)	0.41584 (17)	0.0095 (3)	
O3	1.25301 (15)	−0.0641 (2)	0.33789 (13)	0.0126 (2)	

### Atomic displacement parameters (Å^2^)

**Table:**

	*U*^11^	*U*^22^	*U*^33^	*U*^12^	*U*^13^	*U*^23^
C1	0.0081 (7)	0.0100 (8)	0.0122 (7)	−0.0022 (6)	0.0020 (6)	0.0014 (6)
Cl1	0.01094 (19)	0.0138 (2)	0.0150 (2)	−0.00495 (14)	0.00488 (13)	−0.00150 (14)
C2	0.0113 (7)	0.0081 (7)	0.0102 (7)	0.0005 (6)	0.0016 (6)	0.0013 (6)
O2	0.0121 (5)	0.0133 (6)	0.0163 (6)	−0.0014 (4)	0.0075 (5)	−0.0045 (4)
C3	0.0084 (7)	0.0103 (7)	0.0098 (7)	0.0010 (6)	0.0021 (6)	0.0041 (6)
O3	0.0121 (5)	0.0131 (6)	0.0147 (5)	0.0003 (4)	0.0072 (4)	0.0008 (4)

### Geometric parameters (Å, °)

**Table:**

C1—C2	1.349 (2)	C2—C3	1.508 (2)
C1—C3^i^	1.450 (2)	O2—H2	0.82 (2)
C1—Cl1	1.7164 (15)	C3—O3	1.2240 (18)
C2—O2	1.3217 (19)		
			
C2—C1—C3^i^	121.02 (14)	C1—C2—C3	120.71 (14)
C2—C1—Cl1	121.38 (12)	C2—O2—H2	112.9 (17)
C3^i^—C1—Cl1	117.59 (11)	O3—C3—C1^i^	124.53 (14)
O2—C2—C1	122.23 (14)	O3—C3—C2	117.19 (14)
O2—C2—C3	117.05 (13)	C1^i^—C3—C2	118.27 (13)
			
C3^i^—C1—C2—O2	−178.48 (14)	O2—C2—C3—O3	−0.8 (2)
Cl1—C1—C2—O2	0.7 (2)	C1—C2—C3—O3	−179.74 (14)
C3^i^—C1—C2—C3	0.4 (2)	O2—C2—C3—C1^i^	178.55 (13)
Cl1—C1—C2—C3	179.51 (11)	C1—C2—C3—C1^i^	−0.4 (2)

Symmetry codes: (i) −*x*+2, −*y*, −*z*+1.

### Hydrogen-bond geometry (Å, °)

**Table:**

*D*—H···*A*	*D*—H	H···*A*	*D*···*A*	*D*—H···*A*
O2—H2···O3^ii^	0.82 (2)	2.00 (2)	2.7516 (15)	152 (2)

Symmetry codes: (ii) −*x*+5/2, *y*−1/2, −*z*+1/2.
